# Taking active steps: Changes made by partners of people with multiple sclerosis who undertake lifestyle modification

**DOI:** 10.1371/journal.pone.0212422

**Published:** 2019-02-28

**Authors:** Sandra L. Neate, Keryn L. Taylor, George A. Jelinek, Alysha M. De Livera, Chelsea R. Brown, Tracey J. Weiland

**Affiliations:** Neuroepidemiology Unit, Melbourne School of Population and Global Health, University of Melbourne, Victoria, Australia; University of Birmingham, UNITED KINGDOM

## Abstract

**Background:**

Multiple sclerosis (MS), a demyelinating condition of the central nervous system with an unpredictable course, has a major impact on the lives of people with MS. Partners of people with MS may be significantly affected by the diagnosis, management and uncertainty around disease progression and may provide substantial support and care. Modification of lifestyle risk factors in conjunction with standard medical management has been associated with improved physical and mental quality of life. Adopting major lifestyle modification may have a multi-faceted impact on the person with MS and their partner. Experiences of partners of people with MS have been previously explored, but the experiences of partners of people with MS who adopt this strategy have not. As part of a larger study that aimed to explore partners’ lived experiences of and attitudes towards MS and lifestyle modification, this study reports the active steps and significant changes partners undertook to assist the person with MS and, at times, to also modify their own lives.

**Design:**

Within an interpretive framework, using Heidegger’s phenomenological philosophy, a qualitative study of semi-structured interviews was conducted.

**Participants:**

Aged greater than 18 years and in a spousal relationship with a person with MS who had undertaken an intensive residential lifestyle educational intervention promoting healthy lifestyle.

**Results:**

Themes identified were: adjusting to lifestyle modification, understanding motivations and practical aspects of adjustment; seeking knowledge and support, exploring the ways partners sought positive support for themselves and the person with MS and abandoned negative influences; and embracing well-being, commitment and change, describing the major changes that partners made to their lives professionally and personally.

**Conclusions:**

The experiences of these partners provide clinicians with insight into potential motivations and outcomes of lifestyle modification and suggest potentially positive aspects for those directly and indirectly affected by MS.

## Introduction

Multiple sclerosis (MS) is a chronic neurological condition characterised by an unpredictable neurological decline. The negative impact of MS on people with MS and their partners and caregivers has been extensively investigated. Negative effects for partners include anxiety and distress that may commence at initial diagnosis, and may continue throughout the illness even prior to the onset of significant disability.[1–3] Importantly, quality of life of partners may be negatively affected particularly for those in a spousal relationship, where the duration of MS has been over a longer period, the course of the MS is deteriorating or unstable,[4] and for partners of those with neuropsychiatric disturbance, especially cognitive impairment and depression.[5] Psychological burden is high[6] and health related quality of life is lower in partners of people with MS when compared with controls, especially partners of those with depressive symptoms.[7] Partners are therefore affected by multiple aspects of the person’s illness, but particularly so by their physical and mental health outcomes.

Those partners in care-giving roles may experience an even greater impact on quality of life with higher rates of physical health related concerns and higher levels of psychological distress than the background population.[8] Partners caring for those with disability may experience multi-dimensional carer burden, a response to the combined effects of the physical and psychosocial stressors of caregiving.[9, 10]

Positive outcomes for partners of people with MS, such as strengthening of the relationship through facing challenges together have been reported.[11] Other potential benefits for partners include personal growth from dealing with and adapting to illness; personal health gains through lifestyle modification to ensure the ability to continue to provide care; increased compassion; improved relationships and communication; a change in life priorities and personal goals; and spiritual growth.[12, 13]

The incidence and burden of disease of MS and other chronic diseases are creating new responsibilities for patients, their families and their health care professionals (HCPs). There is a need to shift the paradigm away from the patient being a passive recipient of care to becoming an active partner, applying the knowledge gained from HCPs,[14] and to change the scope of outcomes sought to include physical and emotional function, personal health perceptions and quality of life, rather than just symptom control or cure as in acute disease.[14] This patient centred health care paradigm, where a 'disease-centred' approach is replaced by a 'patient-centred' approach, where both the physician and the patient become proactive, has been described as crucial in paving the way towards optimised care in MS.[15]

Our research group developed an intensive residential lifestyle educational intervention (workshop/retreat) for people with MS that recommends a plant-based wholefood diet plus seafood, omega 3 oil supplementation, maintenance of adequate vitamin D levels, smoking cessation, stress reduction techniques and exercise.[16] Observational evidence indicates that such a healthy lifestyle is associated with improved mental and physical health outcomes importantly including a decreased depression risk and decreased disability, factors known to significantly impact on partner quality of life.[17–20] Uncontrolled intervention studies confirm tangible health benefits to people with MS who attended these workshops at one, three and five years post-intervention and that the majority of intervention participants adhere to lifestyle recommendations most of the time at a three year time point.[21, 22]

Considering that the recommended lifestyle modifications are substantial and outcomes for people with MS may be improved, the aim of the study was to understand if the exposure of the person with MS to the lifestyle educational intervention influenced the partner’s perceptions and experiences of life. We aimed to understand whether the partner experienced any personal or other benefit or any negative effects, as it is possible that the experiences of this particular group of partners may differ from those currently published.

As part of a larger qualitative interview study, we have previously reported one major overarching theme arising from the data, that is, the psychological shift that partners experienced due to the impact of MS and lifestyle modification.[23] In contrast to the psychological changes experienced by partners, this study explores the second overarching theme from the study, that is, the active steps that partners had taken to assist the person with MS and improve the well-being of themselves and/or the person with MS.

## Methods

### Study design

Within an interpretive framework, using Heidegger’s phenomenological philosophy where interviewers bring their own experiences and preconceptions to the research,[24, 25] a qualitative study of semi-structured interviews was conducted.

### Sampling strategy

Purposive sampling was undertaken to select participants according to the research question, that is, partners of people with MS who had attended a lifestyle educational intervention who were willing to explore their experiences and were from diverse enough backgrounds to share varied and unique experiences.[24] A secondary source recruitment strategy was used. Of a research dataset of approximately 2500 online participants (Health Outcomes and Lifestyle In a Sample of people with Multiple sclerosis, the ‘HOLISM’ dataset) approximately 350 people in the dataset had attended a lifestyle educational intervention (LEI) in Australia, New Zealand, the United Kingdom or Europe between 2002 and 2016.[26] Of these 350 people, approximately 80% (280) were partnered and met the eligibility criteria described in the next section.

Using a Microsoft Excel random number generator, these 280 potential participants were randomised. Invitations to participate in the research were then sequentially sent by email in groups of 10. When responses had been received and interviews organised at the participant’s convenience, another 10 invitations were sent. Emails were not sent simultaneously to all 280 potential participants to avoid both participants needing to wait to be interviewed and the non-interview of willing participants once an adequate sample had been achieved. Towards the end of the study, some further purposive sampling of partners of males with MS was undertaken to ensure their views were adequately explored and understood. Sampling ceased when researchers determined that data saturation had occurred. Data saturation is one of four models of saturation which refers to ceasing interviews when researchers identify that nothing new is apparent, and hear the same comments again and again.[27]

The email contained a plain language statement explaining to the person with MS that the study was about their partner. The person with MS was requested to forward the email to their partner that contained a link to a SurveyMonkey questionnaire. The partner was asked to read the invitation and respond to the questionnaire. The first survey question was an invitation to participate. If the partner responded “no” to the invitation, they were directed to the last page of the survey, which thanked them for their time. If partners responded “yes” they were asked to complete demographic data, contact details and the name of the person with MS, so that researchers could determine who had responded, to prevent reminder emails being sent.

### Participants

Eligible participants were aged over 18 years and were English speaking. They had been in a spousal relationship for greater than 12 months with a person with MS who was enrolled in the HOLISM study and who had attended a residential LEI in Australia, New Zealand, United Kingdom or Europe. To be an eligible participant in this study, the exposure required was to be the partner of a person with MS who had attended an LEI. The sample included partners who both did and did not attend the LEI, did or did not adopt lifestyle modification themselves to support their partner, partners of both genders, partners of those with varying duration of illness and degree of disability, and those from both Australia and overseas. Twenty-one partners were interviewed (see Results).

### Interview team

Interviews were conducted by one of two female specialist medical practitioners (SN or KT) working in academic roles. Both interviewers had extensive training and experience in conducting interviews with patients and families for both clinical and research purposes. The interviewers had individually, but not concurrently, delivered the LEI. Interviewers had knowledge of multiple sclerosis and the intervention delivered at the LEI. Partners were asked to identify if either of the interviewers had facilitated the LEI that the person with MS had attended (if they knew) and whether they had attended the LEI themselves. To ensure that the interviewer did not have a pre-existing relationship with the interviewee, an interviewer who had not facilitated the relevant LEI conducted the interview after contacting the partner by email to arrange a convenient time. Participants were informed in the written material that the interviewer was a researcher from the University of Melbourne who had been a facilitator of the LEI.

### The interview

As well as having agreed to the interview on the SurveyMonkey questionnaire, verbal consent to participate was recorded. An introduction, to add context for the participants, and to acknowledge the researchers’ interest in the research question and potential preconceptions, was given: “the reason we are speaking with you today is that current research into partners of people with MS generally paints a fairly negative view of the experiences of people with MS and their partners. We are interested in speaking with partners of people who have been to a residential lifestyle workshop and modified their lifestyle to manage their MS, to understand your particular experiences. Is there anything you would like to ask about the study and me before we commence?”

To supplement data obtained in the questionnaire, some further demographic data was collected. To assess the level of disability of the person with MS, participants were asked “has the person with MS used a walking aid in the last 6 months?” This corresponds with step four or above on the Patient Determined Disease Steps scale, meaning that some form of mobility support was required at least some of the time.[28]

Interviewers followed a pre-designed interview schedule (S1 File). Three broad questions were asked regarding the effect that MS and lifestyle modification had on the partner’s life, their relationship with the person with MS and on their view of the future. Interview prompts were provided for the interviewer but participants were encouraged to expand as much as they desired. Interviews were conducted between July and October 2016 via telephone or internet interface (Skype). Interviewers were located in Melbourne, Australia. Interviewees were located in their home and were generally alone, although two participants indicated the person with MS could hear the discussion but played no role in the interview. No pilot interviews were conducted. Following the conduct of four interviews, interviewers listened to audio recordings of each other’s interviews and were satisfied the interviews were being conducted similarly across interviewers. No adjustments to interviewing technique were required.

### Data collection, storage and transcription

All interviews were audio recorded digitally. Interviews were de-identified, allocated a unique research number and sent to an independent company for transcription. Interviews were transcribed with all names removed. Participant information, digital recordings and de-identified interview transcripts were stored securely and password protected. Interview transcripts were edited by one of the interviewers (SN) while concurrently listening to the audio recordings to ensure accuracy of transcription. Interview transcripts were not returned to participants.

### Data analysis

Interview transcripts were imported into NVivo software. Two authors (SN, KT) analysed transcripts and began coding the data as interviews were recorded. Although two researchers acknowledged preconceptions, no *a priori* thematic framework was established, but rather themes were data-driven, emerging from the content of the interviews. Researchers met regularly to discuss codes derived from the data. Interviews were reread, re-coded and emerging themes identified and revised until researchers felt they adequately reflected participants’ experiences.

Four overarching themes from the initial data were identified of which “Taking Active Steps” was one. When identified as such, a further search for themes and subthemes within “Taking Active Steps” continued and chosen verbatim quotes were then reviewed to ensure themes reflected the raw data.

### Trustworthiness and reflexivity

Researchers developed rapport with interviewees during interviews and familiarised themselves with the data by frequent re-reading of transcripts. The researchers therefore had a high level of familiarity with the data. A third researcher, a practising clinical psychologist (TW) who had not facilitated the LEI, reviewed the data with the interviewers at several stages, querying the coders to assist them in development of initial codes, interpretation of data, the search for themes, development of a theme hierarchy, theme naming and reaching a final consensus on themes. The familiarity with the data and the presence of multiple researchers reviewing and analysing the data and determining emerging themes added to credibility and confirmability[29] of the data. A journal documenting research discussions and theme development was kept.

Reflexivity[30] was addressed as SN and KT, having had extensive personal experience with attendees at LEIs, acknowledged their preconceived beliefs regarding the potential experiences and benefits to partners of people with MS who had attended an LEI. TW also assisted in promoting dialogue and discussing the potential influence of these preconceptions between researchers.

An audit trail is provided in S2 File. Consolidated criteria for reporting qualitative research (COREQ) reporting guidelines[31] are provided in S3 File.

### Ethics

The study was approved by the University of Melbourne Human Research Ethics Committee (ID number 1545280.1). Participants provided consent to interview in the on-line survey that they completed and consent was also recorded at the commencement of the interview.

## Results

Of the potential 280 participants in the dataset, 103 email invitations were sent before recruiting ceased. Twenty (19%) invitees formally declined participation. Seventeen (17%) declined by completing the survey by selecting “I do not wish to participate”. No free text area to provide a reason for declining participation was provided as researchers felt that those who did not wish to engage with the research project should not be asked for further information. Three (3%) declined by return email directly to the researchers, two (2%) indicated they no longer had a partner and one (1%) had never been to a retreat. Fifty-nine (57%) email invitations to participate did not receive any response by email or SurveyMonkey. Twenty-four (23%) partners accepted the invitation to participate but three (3%) were unable to be contacted following the return of their surveys.

Demographics of the study participants have been previously described in detail.[23] Twenty-one (20% of invitees) interviews were conducted, six women and 15 men. Duration was between 20 and 62 minutes with an average of 36 minutes. Ages of participants ranged from 28 to 79 years, duration of spousal relationships from 5 to 51 years, and all relationships were heterosexual. Characteristics of the participants are described in Table 1. Of the people with MS, duration of MS ranged from approximately two years to greater than 40 years and six had a mobility disability defined as using a walking aid within the last six months (including one person using a wheel chair).

**Table 1:** Participant (partner) demographics.

Variable	Number (% approximate)
**Sex**	
Male	15 (71)
Female	6 (29)
**Age (years)**	
20–29	3 (14)
30–39	2 (10)
40–49	4 (19)
50–59	3 (14)
60–69	7 (33)
70–79	2 (10)
**Place of residence**	
Australia	8 (39)
New Zealand	6 (28)
United Kingdom	6 (28)
Europe	1 (5)
**Duration of relationship (years)**	
5–10	7 (33)
11–20	3 (14)
21–30	4 (19)
31–40	2 (10)
41–50	4 (19)
>50	1 (5)
**Attendance at LEI with person with MS**	
Yes	11 (52)
No	10 (48)
**Years since LEI attended by person with MS**	
1–2	5 (24)
2–5	7 (33)
>5	9 (43)
**Employment**	
Part/full time	13 (62)
Retired	6 (28)
Unable to work	1 (5)
Maternity leave	1 (5)

MS: multiple sclerosis; LEI: lifestyle educational intervention (workshop/retreat)

The lifestyle modification program is commonly referred to as the Overcoming Multiple Sclerosis program (“OMS program”). The program is described in the book Overcoming Multiple Sclerosis–the evidence based 7 step recovery program[16] and is the basis of the LEI. The recommended diet is an ultra-low saturated fat plant-based wholefood diet plus seafood and participants often refer to it as the “OMS diet”. The lifestyle educational intervention is often referred to as “the workshop” or “the retreat”. The gender of the interviewees and their research number are indicated following each quotation as (F/M and P#). All of the names of people with MS are indicated as [P/partner]. Repetition, informal language and exclamations were deleted. Any deletions from the verbatim quote are indicated as …. Explanatory notes are included as (…).

Some themes emerged from varied expressions from multiple participants while other themes related to very specific expressions from some participants. It was therefore not possible to quantify the proportion of participants expressing each theme. Quotations that were thought to best represent the essence of each theme were selected for inclusion in the manuscript, and are indicated with italics.

Three major themes under the umbrella theme of ‘Taking active steps’ were identified:

1. Adjusting to lifestyle modification
2. Seeking knowledge and support
3. Embracing well-being, commitment and change.

Subthemes are described within the text.

### 1. Adjusting to lifestyle modification

#### Making changes to provide support

While degree of adherence to lifestyle modification by people with MS or adoption of modifications by partners was not assessed, the theme of adjusting to lifestyle modification reflected how partners initially adjusted to the changes the person with MS was making and why, and reflected their degree of willingness to make changes themselves. A desire to support the person with MS was a major motivation for partners choosing to make changes. Adopting lifestyle modification along with the person with MS was experienced as challenging but as a way to provide practical support towards adopting and maintaining modifications. Active participation in the changes by the partners also provided emotional support to the person with MS and benefit was experienced through facing challenges, the enjoyment of participating and making changes together and achieving goals.

*It was a challenge and I think we went through the same challenges that everybody does that's doing it*, *but it was easy in the fact that once we'd made it through the first four weeks we…came out the other side and we've been on the diet now for six years without any challenges*. *(M*, *P21)*

*Participant*: *We challenge each other in meditation through*, *I think it's called the UK Headspace app*, *which we both do*. *So you can kind of see how the other one's performing*. *Interviewer*: *A meditation competition to see who's the most chilled out*? *Participant*: *Exactly*! *(M*, *P4)*

*We've always been quite into exercise*. *I've always been a cyclist and cycling's something that my partner has taken up since she was diagnosed*. *So I cycle with her a lot*, *as much as we can*. *(M*, *P16)*

The support and encouragement provided by families further enabled the couple to establish, adjust to and maintain change.

*The really good thing too is our whole family has embraced that*. *So if we go and have dinner with my sons or my sister or whatever*, *they’ve always got food that my partner can eat*. *The whole family just made good changes in that respect*. *(F*, *P3)*

#### Making changes for potential personal and family benefit

Further to a desire to provide support, at times partners’ motivation evolved into a desire to improve their health of themselves and their families, when they recognised the potential for personal or family health benefits achieved through dietary modification in particular. Benefit was experienced from the experience of tangible benefits to the partner’s own health.

*Mainly I started to so that I could just support [partner] on his quest to see if it did anything for him*, *if it worked*, *if he got better*. *But right away we both saw the benefits*. *I strongly believe in a diet for MS but actually*, *if you look at it*, *personally I think it's a good all round diet for just about anyone*. *(F*, *P17)*

*I followed everything as well*. *The only difference at the outset*, *I kind of had a little cheat meal*, *which was if we went to the pub to watch the football and I would have a chicken parmigiana… until we went to the retreat*. *That's when I was like well this is good for my health as well*, *so then now we both follow it to the letter… Then also I guess I lost a significant amount of weight*. *I wasn't overweight to begin with but just through the amount of fats that I was eating in my day-to-day life I ended up losing 15 to 20 kilos*, *just from doing the dietary lifestyle changes*, *and of course exercising and getting more vitamin D and all of the above*. *I guess it was all positive things (M*, *P4)*

Multiple motivations to adopt change were sometimes present. Associated with benefit to the person with MS and potential health benefit to self and family was the added benefit of managing the practicalities of daily life when the whole family adopted change. Partners spoke of the benefits of everyone being on the “same page” when managing meals and other aspects of daily life.

*We have two daughters*. *So*, *based on the statistics*, *we decided that both [partner] and the girls would go on the OMS diet and if 75 per cent of the house was doing it*, *it makes sense that we all did it*. *So*, *I changed and went onto the OMS diet as well*. *So*, *we just got rid of everything in the house that wasn't part of the OMS diet and changed as a family*. *(M*, *P21)*

*But now it's just part of—we don't even think about it*. *I now follow—I don't eat meat or dairy either now mainly because it just—it was easier to just cook one meal and also it's hard to read all this research and then continue putting that stuff in your body I found*. *(P20*, *F)*

*To be honest*, *I think making those changes for a person with MS makes everybody's life more healthy in many respects*, *in terms of the diet*. *It's like making modifications with anyone with a disability*. *It tends to help everybody at some level*. *Certainly*, *I'd encourage people to think about the health benefits of it*. *(P3*, *F)*

#### Finding one’s own approach

Within this subtheme, the motivation for and degree of change evolved over time as partners considered and evaluated their own needs and wants with respect to the choices they made about lifestyle modification. Support for the person with MS did not change, but partners found their own way forward with diet, exercise and other modifications. Partners considered their own needs and health imperatives and made personal choices regarding their degree of engagement with lifestyle modification.

*Immediately after the retreat our diet changed considerably*. *So [partner] has pretty much gone vegan plus fish exclusively and she went cold turkey on meat*, *so to speak*, *which has been a bit of adjustment for me*. *So*, *I've been a meat and three veg guy for 35 years*, *so it's hard to adjust to that*. *I haven't given away meat*, *but it's obviously added some complexity to arranging dinner and all this sort of thing*. *So*, *my diet has changed accordingly*, *but maybe I'll throw in meat or something on top of a vegetarian meal*. *(M*, *P19)*

*Yeah it's my choice*, *and I don't need to do it as stringently as [partner] does*. *[Partner] is religious with it almost*, *he follows it completely*. *It helps him mentally as well as physically I think*. *Myself*, *yeah I do stray but overall I really like the diet and I think the whole non-meat*, *and not fried and everything is still healthy for you*, *you can't argue with it*. *(F*, *P17)*

*It's been a process*. *I mean it was difficult to get into the meditation and… we've not done it 100 per cent from the beginning*. *Even now [partner] still has his moments*, *and there are still things he can improve on*, *there always are*. *It's the fact that it's a lifestyle thing so you don't need to do it all in one go*. *You can do it gradually and adapt your life to it as the years go by*. *(F*, *P7)*

#### Barriers to adopting changes

Despite positive attitudes toward change and strong motivations to assist and adopt changes, barriers to making changes were experienced. Barriers were practical, such as eating out, travelling, and adopting changes into every day life when major life events occurred. Others experienced challenges to their motivation and emotional barriers when MS progressed and the tangible rewards did not seem as obvious. Partners reflected on the barriers that the person with MS faced and consequently how they experienced those barriers.

*You’re a natural outcast (in France) if you don't have things cooked in butter*. *Italy was a dream but France was a disaster*. *(M*, *P2)*

*It was really good until (the baby) was born and then things get a bit harder*. *We were in almost a strict wholefood diet prior to that*. *There are shortcuts having to be taken because of the amount of time that we've got*. *But there is a plan to get back on track with that as well*. *(M*, *P19)*

*So she's still a fish-eating vegan but* … *she'll have a bit of cheese which previously she wouldn't have had…she didn't know if it did her any good or not*. *I suppose that's the problem with all this stuff you just don't know*, *but she thought she'd rather have a slightly more enjoyable life*. *We were in France earlier on in the year so we hit a few crème cakes and things that probably weren't on that strict diet*. *(M*, *P13)*

### 2. Seeking knowledge and support

#### Broadening knowledge and understanding

This subtheme encompassed a desire by partners to strengthen the resolve of themselves and the person with MS to continue to improve their health by seeking knowledge and evidence. Partners demonstrated enthusiasm and rigour in undertaking their own research and obtaining further knowledge that supported the changes they were making. Seeking knowledge on behalf of the person with MS was viewed as a way of supporting the person. Other partners saw reading the literature as a way of protecting the person with MS from becoming disheartened by negative literature.

*I read a lot of books… because I wanted to know everything there was to know about it*. *So [partner] and I made the decisions together—because his father had obviously suffered from MS*, *it was something that was very close to [partner's] heart*. *It's a bit more difficult to read the literature in the sense that it's a constant reminder of what his father went through and also it can be a bit depressing because not all the books are positive*. *So I read all the books and I will only share with him information that's relevant to us*. *So that's what I did*. *(F*, *P7)*

*We both like to cook*. *So we do research*, *do recipes and so on or we look to adapt maybe non-MS friendly*, *we call them*, *recipes to make them compliant*. *So yeah*, *it's another area of interest really that we've worked on together and we've developed together*. *(M*, *P5)*

#### Establishing positive relationships

Being surrounded by positivity through actively choosing to establish and continue relationships with like-minded people who supported the choices and actions of the couple were important for partners. For those who had attended the workshops, the sense of community that resulted from these connections was a support that many sought to continue following the retreat. Some families made changes to their diets and lifestyle to be inclusive and provide tangible assistance by normalising the dietary change within the family. Such encouragement provided by families and friends further enabled the couple to establish and maintain change.

*The community that comes out of the thoughtful patients and their friends is much more valuable*. *I'd see that as one of the big benefits of the retreat*, *is actually helping to create that group*. *(M*, *P14)*

*The really good thing too is our whole family has embraced that*. *So if we go and have dinner with my sons or my sister or whatever*, *they’ve always got food that my partner can eat*. *The whole family just made good changes in that respect*. *(F*, *P3)*

*[Partner’s] mother has made dietary changes herself as a result of going to the retreat*, *just as general wellbeing*. *She's a big supporter of [partner] and she's probably the person that [partner] would consult with the most as far as if she's feeling a bit down about how things are; I've got to get my diet back on track*. *She would actually ring her mum and say I'm struggling here*, *then get a bit of a pick-me-up through that*. *(M*, *P19)*

Seeking support from health care professionals (HCPs) was a high priority. As for most people with a chronic condition, most people turned to their health care professional for support. Interaction with the medical profession and associated healthcare professionals had a significant impact on the person with MS and the partner. A supportive, nurturing professional relationship with health care professionals added to the support provided by family and friends and was highly valued. Positivity and encouragement from HCPs added to the sense of a circle of support.

*She's got a very good local doctor*. *On top of that she goes for—I think it's every six months she goes to see her specialist*. *On top of that we have been on another small get together for people that suffer from MS*, *where we just had a series of—I think it was six meetings on a Wednesday night*, *just any other couples and other people that have been affected by it*. *(M*, *P6)*

*The GP is very supportive and very encouraging*. *(M*, *P9)*

*The reason I say that is because every time we go and see [neurologist] [partner] takes along all the latest research and they talk about it together*. *He says*, *I don’t know anything about that*, *I’ll look it up*. *So again*, *a neurologist that is prepared to take that all on board and do some work on it is quite fantastic*. *(M*, *P18)*

#### Abandoning negative influences

In this subtheme, partners expressed the undermining nature of negative interactions. These interactions could be on-line, personal relationships or, at times, relationships with HCPs. Partners described feelings of loss of hope associated with these interactions. Negative interactions and messages received on the internet often led to couples “unplugging” themselves from this potential influence. Friendships re-ordered themselves, as those friendships that provided support and encouragement were valued, and those that did not were re-evaluated.

*(We) pretty much unplugged ourselves from a lot of the internet forums and a lot of the negativity that was out there*. *I guess for us now*, *six years later…we're not really plugged in too much*. *(M*, *P21)*

*He's addressed—which might be quite sad—but friendships that he had that weren't necessarily positive for him… just trying to readdress what he needs in his life and what is good for him*. *(F*, *P20)*

Relationships with HCPs at times did not provide the support that the couple was seeking. There was a sense of being stuck in the middle of a “tug of war” between approaches to managing their health. At times, the interactions were characterised by a lack of constructive advice and care. But further to that, the couples perceived that their attempts at self-care and self-management were unsupported and, at times, criticised and undermined. Experiences were sometimes so unhelpful that the professional relationship, and potential care and support, was abandoned.

*But it's interesting watching the medical profession. Again, I get a bit cranky there because you think oh come on*, *it's not a bloody tug of war. (M, P13)*

*We haven't seen a neurologist for about seven years now. When we did go with that small relapse about three years after the program, he basically said I've been waiting for you with all that rubbish you do. So we haven't seen him since*. *(F, P1)*

*(The neurologist) was so inconsiderate and the way he handled the diagnosis and the way he told us, it was horrible. It was… very negative…He basically said to [partner], you need to stop this (the OMS program) now. You need to do the disease modifying drugs. You are putting your family in jeopardy*. *(F, P7)*

### 3. Embracing well-being, commitment and change

#### Embracing the whole program

After sometimes tentative beginnings to adopting change, and consideration of the partner’s own desires and needs, motivations sometimes progressed to partners deciding to embrace the program for their own reasons as well as to support the person with MS. Taking the step of adopting change wholeheartedly added a dimension to the partners’ lives of having made their own changes that were positive and were themselves.

*So, the conversations that we were having were not just about MS and the benefits that it brings to [partner] but also the benefits that it brings to me… in terms of being healthy and reducing my risks of heart disease and cancer and all of those other things… We were very early in the journey for [partner's] MS and we didn't know where that was going to go, so the last thing that she wanted was me being unfit and sick and having problems when she was potentially struggling with her health. How were we going to raise two girls if we've both got health issues? So, for me it was about me being in the best health that I can be as well*. *(M, P21)*

*When I first started OMS (the program) I lost 35 kilograms. So, for me personally it was a massive lifestyle change and benefit too—it was incredible. I don’t worry about my health if I'm on OMS. I would have been a very sickly person as it was but I'm at my healthiest when I'm on the program. I saw that especially when I was pregnant and I came off the program and the way my body felt and how—I really struggled through pregnancy and I'm convinced it's because I started eating meat and dairy. Not just meat and dairy*, *meat, dairy—everything bad. (F, P7)*

#### Developing commitment

Similar but further to embracing change, was the development of commitment to adopting the lifestyle. This commitment to change was seen as the key to the success of the person with MS and themselves in being able to adopt and maintain a healthy lifestyle. These partners saw these outcomes as being reliant on making changes with positivity.

*My advice would be try and, as a supportive partner, be as close to living it with your partner. It's hard enough for them accepting that they've got MS as well as having to then completely change their lifestyle in terms of what they eat and all of those things. So, if you… can make that as less stressful as possible, that's where you're going to see the success*. *(M, P21)*

*I would say that jump into the approach 100 per cent*. *Don't hold back*, *just really get in there and really explore the exercising and the diet and the meditation and really just enjoy it because it will completely change everything for you*. (M, P9)

*So, we are where we are with [partner] and we're doing everything that we can for her today. But at the same time, we're also doing this for our two daughters for tomorrow*. *(M, P21)*

#### Making major changes

Apart from adopting the key lifestyle modification factors such as diet, exercise and stress reduction, some partners made major changes to their lives to reduce stress and allow more time together and to live a more manageable lifestyle. These partners chose to make changes proactively, rather than with any sense of compulsion or as a response to adverse circumstances that required re-evaluation of employment or housing etc. Major steps were taken to modify to careers and places of residence to allow them to live the life they wished to live.

*I could have continued on with my career and my lifestyle but we both made a decision to use the MS, not as a negative like it always had been, but start looking at it as a positive and… just completely change our lifestyles. So that's why we sold the house there and I quit my work and bought a house that needed a lot of renovations and now run a bed and breakfast, so that we have more of a self-sustainable lifestyle and a step back from that sort of rat race … It's very hard work, I absolutely love it. Because it's given us the lifestyle that I think is best for both of us. It gives me a chance to be with [partner] more…so this is enabling us to take both of our lives in our own hands and try to do something alternative to help [partner], and myself*. *(F, P17)*

*We made the decision for him to quit so he works from home now and we work together, and that's made a huge difference. [Partner] was a teacher. He has now opened up his own business. I was a teacher… and now I have started working with [partner]. Our lifestyle is something we changed. We work from home. We manage our own stress. We are each our own bosses. We actually are so much happier with our new job roles than when we were teaching which is not what we expected*. *(F, P7)*

*I worked in finance (overseas) and that's quite stressful; long hours and it's quite cutthroat. So coming to (Australia) was part of a de-stressing exercise really…Life isn't as stressful as it was; we have a good work/life balance*. *(M, P5)*

## Discussion

As part of a larger study that examined the experiences of partners of people with MS who had attended a workshop recommending healthy lifestyle, this study examined the motivations, decisions and active steps taken by partners of people with MS both as a support to the person with MS and for themselves as the couple faced the challenges of developing a healthy lifestyle.

With respect to adjusting to lifestyle modification, some partners embraced the changes the person with MS made immediately, adopting the diet, exercise and other lifestyle factors. The behaviour change was seemingly easy as they recognised potential benefits to the health and well-being of the person with MS and themselves. These partners applied themselves to adopting change with “grit”, where they developed a passionate interest in the lifestyle and adopted change with determination and effort.[32] Passionate internalisation of an activity into one’s own identity, is a predictor of the ability to make deliberate change [33] and for those who adopted change in this manner, their passion was expressed. For others the process involved the development of “self-control”, where there was an initial conflict between two goals (wanted to eat chicken parmigiana versus supporting the partner) but there was a gradual shift toward the more valued longer term goal,[32] and at times, there was a combination of both determination and self control required to achieve change.

For others there was a process of adapting to the changes over time and some found their enthusiasm and ability to maintain change waxed and waned, finding their own way over time, especially with respect to diet and nutrition. Without the personal diagnosis of MS, the health-related imperative did not apply to the same extent, although this perception at times changed as partners recognised their own health needs and the potential personal physical and psychological gains of lifestyle modification. There was an evolution in perspectives from viewing lifestyle changes as something being imposed externally to something that was willingly and wholeheartedly embraced. Changes made were reinforced by the knowledge obtained from further research, the benefits experienced to partners’ own health and life, and making the changes a habit and incorporating them into the routine of their daily lives.

A major theme was the steps partners took to access resources and seek support. Some partners took a very focussed, academic approach and sought greater knowledge from the medical literature and internet sources to underpin their actions. Seeking assistance from family and friends was also described and there was often an element of surprise expressed about the lengths to which others went to assist them in their attempts to make changes and to be inclusive by making changes to show their support. Although support from others is well known to benefit partners of people with MS,[34] the support provided by friends and family in these circumstances was more than just emotional support, and extended to others making significant behavioural changes themselves to support the couple.

Some sought support networks beyond family and friends and valued these connections. The workshop was seen as a great resource for developing and maintaining such support networks. Attendance at the LEI has been shown to improve health related quality of life,[35] but the degree of continuing engagement with the resources (support groups, books, websites, forums) is also important and confers added advantages. Engagement has been found to be associated with a clinically significant reduction in fatigue and depression risk, and significantly better physical and mental health-related quality of life scores compared with no engagement[36] once again leading to better outcomes for partners. Group support for a lifestyle modification program in obesity has also been effective, importantly not only to the desired outcome of successful weight loss, but in developing an improved sense of self efficacy, greater friend support and better quality of life and these psychological improvements were sustained post intervention.[37] Therefore, for those actively seeking and engaging with support, the group support for lifestyle modification in MS provided assistance with achieving health related goals and benefits that extend to sustainable improvements in quality of life for the people with MS and their partners.

A very strong theme that emerged was that of abandoning negative influences, especially negative interactions with HCPs. Input into shared decision-making in MS influences satisfaction with medical care, as does open communication between HCPs and patients and families, and a sense of empowerment and participation in decision-making is thought crucial to achieving optimized care in chronic neurological diseases, such as MS.[38, 39] The Australian Commission on Quality and Safety in Health Care (ACQSHC) commissioned a large review of the Australian and international literature regarding patient experiences and found that professional communication, opportunities for patient/carer involvement and the attitudes and behaviour of health professionals were the most important features determining a positive or negative care experience for patients and carers.[40] ‘Denying patient and family involvement’ resulted in negative experiences. Lack of interpersonal skills and professionalism and lack of empathy among HCPs were key characteristics of negative experiences, generally highlighted by vulnerable patient groups.[40]

In this study, where patients and partners were attempting a paradigm of healthcare not yet widely accepted outside the standard medical model, aiming to achieve empowerment and self-efficacy, hope and positivity emerged frequently throughout the interviews as important facilitators of the ability to make and maintain lifestyle changes. Loss of hope and lack of support was experienced as a negative interaction that they wished to remove from their lives. Partners expressed a clear ability to manage without the support of their HCPs in these circumstances, but the negativity of these interactions had a major effect on the person with MS and the partner and resulted largely in the severing of these potentially important and fruitful relationships.

For many, the simple process of initiating lifestyle modification developed over time into a commitment to further embrace lifestyle modification. This change appeared to be one of a shift from being passively to actively embracing wellbeing with a sense of taking control of life and health. Partners described their support of the person with MS in terms of being “on board”. They described rewards both in their own personal gains and in the assistance they provided to the person with MS. Following on from this, many made major changes to improve their lives.

Negative employment outcomes for partners of people with MS have been previously described, and consisted of reduction in working hours resulting in financial hardship[1], resignation, not seeking or declining promotion when offered, changes to lower grade jobs in order to avoid taking more responsibility[41], increased numbers of days off[8] and other negative impacts on work.[41] Some partners did describe making such changes but others chose to pro-actively change their circumstances of employment to improve their lifestyle and to assist in adoption of lifestyle modification, particularly stress reduction, a change not previously described for partners of people with MS. For some, approaching these situations with positivity culminated in a complete change of career, relocation to another part of the country or overseas, or reduction in the amount of time they worked. Others chose to move place of residence to facilitate a different pace of life, more time together and allow time for things they considered more important.

While apparently simple and practical lifestyle modifications were being recommended and made, with the aim of improving health and quality of life, the process of implementing these changes was described as leading participants and the couples to change many aspects of their lives together. Rather than being passive and uncertain, participants noted a sense of control and optimism and tangible benefits to their physical and psychological well-being that came with taking active steps towards improving health. For some, the sense of being on a shared journey ensued, supported by each other, family, friends and social networks that followed attendance at the workshops.

Broadly, the experiences of partners described in this study show that MS and lifestyle modification not only affect the person with MS but also have a significant impact on partners. They highlight that, while partners described many challenges, the process of adopting lifestyle changes by the person with MS led to partners willingly assisting the person with MS, with many also adopting the changes themselves and describing many positive experiences during the journey. Some made small steps and others took giant steps, but generally described enriched lives as a result, in contrast with many findings of outcomes for partners and spouses of people with MS.[42]

A key message that emerged from the study is the value of support. Although lifestyle modification is clearly achievable with no external support, our study and the wider literature demonstrate the value of support of all kinds to the person with the illness and their partner. Clinicians should be sensitive to the adjustments and coping skills of both members of a couple where one has MS[43] and encourage families to take an active role in their health and foster hope and positivity. Equally they should be aware of the harm that they can do through insensitive communication and from discouraging patients and their families from actively participating in a healthy lifestyle for their own care, and eroding genuine hope.

## Limitations

The participants were partners of a select subgroup of people with MS who had undertaken a specific lifestyle educational intervention, generally adopted lifestyle modification and participated in the HOLISM study and were, therefore, likely to be a very motivated group of people. Only a proportion of the 280 potential participants was invited. The researchers did not wish to invite people and then not interview them once sampling ceased. The fact that not all of the potential sample was invited may have introduced some selection bias, but the sample was randomly selected to minimise potential bias.

There may have been response bias as those partners with experiences differing from those described in this paper may have chosen not to respond to the invitation to participate. Those who declined participation were not asked for their reasons for non-participation, as the researchers felt that to ask reasons was intrusive and unethical. The themes expressed are therefore those of the partners of this select group. While the interviewers reflected on and attempted to understand their own preconceptions and biases, and sought to account for these, including by adding an uninvolved experienced researcher to the thematic analysis process, these preconceptions may have had unrecognised effects on the derivation of themes and the assessment of their significance. Participants were all English speaking and all in heterosexual relationships and therefore may not be reflect experiences of non-English speaking or the LGBTI community.

## Conclusion

The themes expressed in this study, those of adjusting to the practicalities of lifestyle modification, seeking support, and embracing wellbeing, developing commitment and making major life changes, represent novel experiences of partners of a select group of motivated patients. These experiences may encourage other partners of people with MS, and those with other chronic illnesses, to consider supporting and adopting lifestyle modification to both assist the person with MS and to find positive outcomes for themselves. A broad range of support can assist in making and maintaining lifestyle change and thereby providing hope and positivity, including that provided by family, friends and importantly healthcare professionals.

## Supporting information

S1 File Interview guide. (DOCX)

S2 File Audit trail. (DOCX)

S3 File COREQ guidelines checklist. (DOCX)
