# Supplementary material for: Taking active steps: Changes made by partners of people with multiple sclerosis who undertake lifestyle modification
Source: PLoS One. 2019 Feb 28;14(2):e0212422. doi: 10.1371/journal.pone.0212422 (PMC6394935; doi:10.1371/journal.pone.0212422)
Supplement: S1 File — (DOCX) [file pone.0212422.s001.docx]

**S1** File. Interview guide.

**Demographics.** Prior to commencing the interview the participants will be asked some essential demographic data:

1. Demographics of interviewee (The Partner)
   1. Age
   2. Gender
   3. Employment status
   4. Location (where do you live)
   5. Duration of spousal relationship
2. Demographics of Person with MS
   1. Age
   2. Gender
   3. Type of MS and time since diagnosis
   4. Level of disability
      1. In the past 6 months has your partner used a mobility aid?
      2. If so, what sort of assistance did they use?
      3. When did your partner attend the lifestyle educational intervention?
      4. Did you attend the lifestyle educational intervention with them?

**Interview**

**There are 3 main questions (dark). Participants will be encouraged to explore and answer the questions in any way they feel appropriate. The prompts (light grey) are only if the required to stimulate conversation.**

1. How has MS and any lifestyle modification that you/your partner have undertaken affected your life?
   1. Has there been little change or significant change?
   2. Have there been positive changes?
   3. Have there been negative changes?
   4. What have been the easiest changes and what have been the hardest?
   5. What barriers have you identified to implementing lifestyle change?
   6. Have other health care professionals (eg GP or neurologist) supported any changes you have made/tried to make?
   7. What other resources have you found useful?
   8. Do you have any tips/advice for others?
2. How has MS and any lifestyle modification that you/your partner have undertaken affected your relationship with the person with MS?
   1. Have there been positives for your relationship due the diagnosis of MS or the changes you have made?
   2. Have there been difficulties in your relationship due the diagnosis of MS or the changes you have made?
   3. What strategies have you found helpful in managing the issues you have described?
   4. How have you dealt with any changes to the relationship?
3. How do you see your future and what has influenced this view?
   1. Your life plans/children/career
   2. Your health
   3. Your relationship
   4. Do you feel in control of your future?
