# Supplementary material for: Taking active steps: Changes made by partners of people with multiple sclerosis who undertake lifestyle modification
Source: PLoS One. 2019 Feb 28;14(2):e0212422. doi: 10.1371/journal.pone.0212422 (PMC6394935; doi:10.1371/journal.pone.0212422)
Supplement: S2 File — (DOCX) [file pone.0212422.s002.docx]

**S2 File.** Audit trail.

The following audit trail is based on that recommended in

Nowell, L.S., Norris, J.M., White, D., Moules, N.J. Thematic Analysis: Striving to Meet Trustworthiness Criteria. *International Journal of Qualitative Methods.*16;1-13

1) Ensuring familiarity with data

- Excel spreadsheet listed all potential participants, date of initial email invitation, reminder, response to email, date of interview
- All records archived and stored securely as de-identified original audio recordings and transcripts
- Initial thoughts of possible emerging themes following first interviews documented
- Researchers listened to each others’ interviews to ensure consistent technique
- Immersion in data by frequent re-reading of transcripts
- Import of data to Nvivo
- Saved serial versions of Nvivo files to both ensure data was saved and to document evolution of themes

2) Generation of initial codes

- Codes were generated as interviews were conducted and reviewed
- Interviews were analysed and quotations of interest were highlighted and allocated nodes within Nvivo software
- Some items were coded within multiple nodes
- Codes discussed among three researchers and reviewed and some codes were aggregated
- Memos of researchers discussions and thoughts were maintained to help keep track of ideas of emerging themes

3) Searching for themes

- “Clusters” of codes were identified and merged into groups that linked significant concepts.
- Researchers determined that overarching themes had become apparent and worked through the data many times until overarching themes that signified concepts that represented the essence of interviews were identified. These themes emerged from the data and were not determined *a priori.*

4) Refining themes

- The overarching themes determined at this stage of analysis were:

| Perspectives and experiences of partners of people with MS | | | |
| --- | --- | --- | --- |
| Psychological shift (the thinking) | Taking active steps (the doing/actions) | On the path together (relationships) | Standing on the edge (views of the future) |

Screen shot Nvivo software (see below) demonstrating emergence of overarching themes. Refinement not completed at this point.


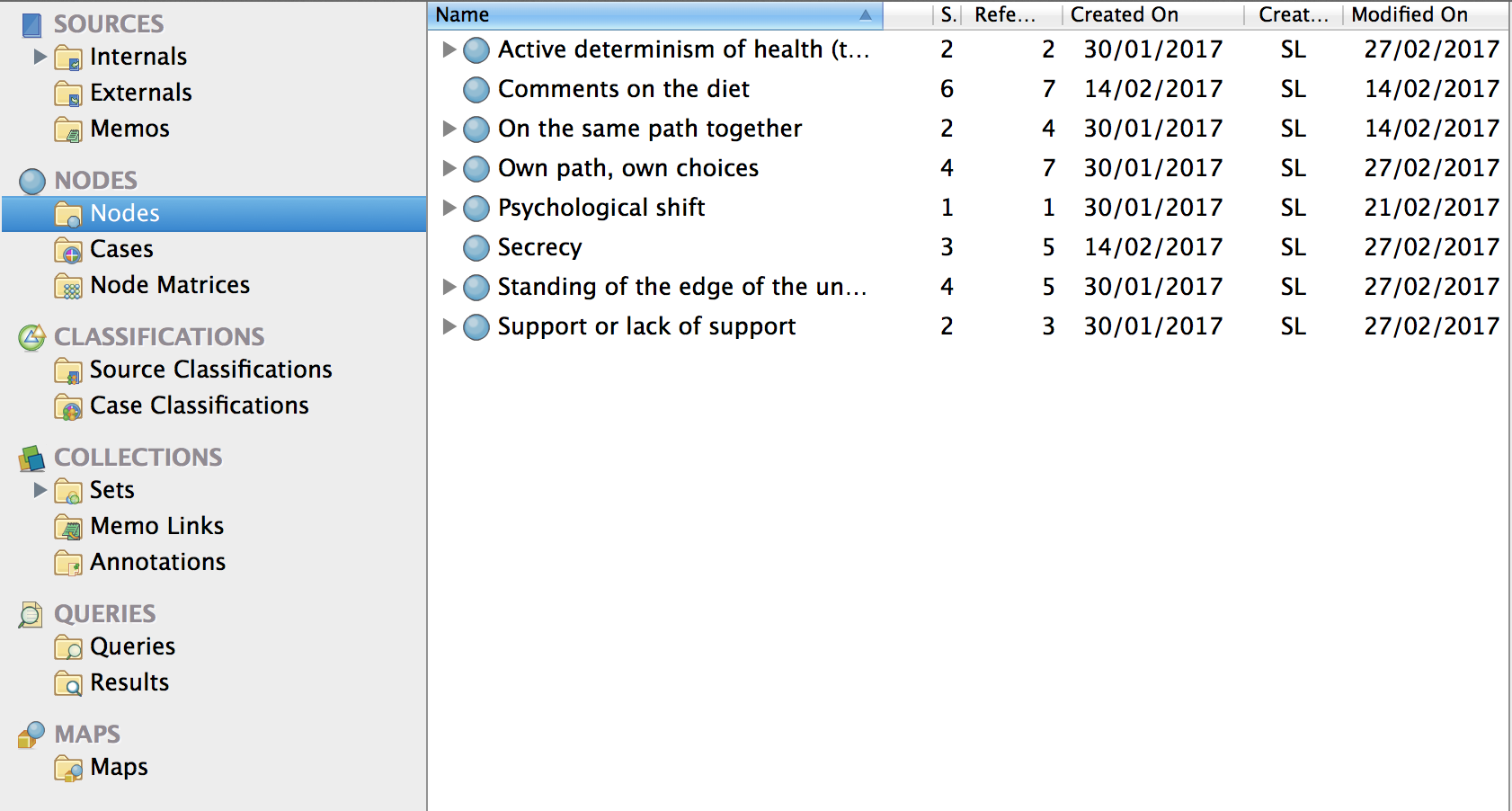


5) Defining and naming themes

- This study analysed the overarching theme of Taking Active Steps (here named as active determinism.)
- Potential themes within Taking Active Steps were categorised as child nodes in Nvivo (See below) and were revised and recoded following re-reading and researcher discussions

Screenshot of Nvivo software demonstrating themes development within Taking Active Steps


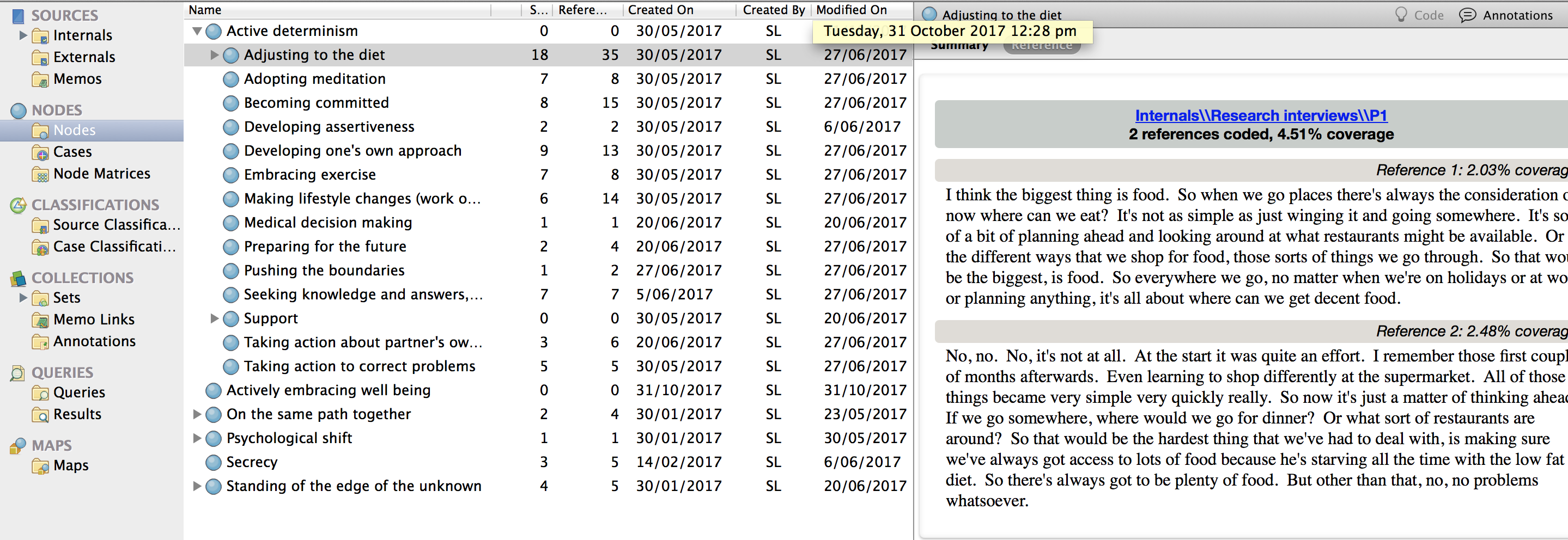


- Themes were reviewed to consider if they represented an important concept arising from the data and were assigned names

6) Producing the report

- Representative verbatim quotes were selected to illustrate subthemes
- COREQ guidelines were utilized to ensure detailed and transparent methodology was communicated (See S3 COREQ guideines)
